# Chronic Renal Transplant Rejection and Possible Anti-Proliferative Drug Targets

**DOI:** 10.7759/cureus.376

**Published:** 2015-11-06

**Authors:** Adnan Bashir Bhatti, Muhammad Usman

**Affiliations:** 1 Department of Medicine, Capital Development Authority Hospital, Islamabad, Pakistan; 2 Department of Medicine, Jinnah Hospital Lahore (JHL)/Allama Iqbal Medical College (AIMC), Lahore, Pakistan

**Keywords:** renal transplant, rejection, drug targets

## Abstract

The global prevalence of renal transplants is increasing with time, and renal transplantation is the only definite treatment for end-stage renal disease. We have limited the acute and late acute rejection of kidney allografts, but the long-term survival of renal tissues still remains a difficult and unanswered question as most of the renal transplants undergo failure within a decade of their transplantation.

Among various histopathological changes that signify chronic allograft nephropathy (CAN), tubular atrophy, fibrous thickening of the arteries, fibrosis of the kidney interstitium, and glomerulosclerosis are the most important. Moreover, these structural changes are followed by a decline in the kidney function as well. The underlying mechanism that triggers the long-term rejection of renal transplants involves both humoral and cell-mediated immunity. T cells, with their related cytokines, cause tissue damage. In addition, CD 20+ B cells and their antibodies play an important role in the long-term graft rejection. Other risk factors that predispose a recipient to long-term graft rejection include HLA-mismatching, acute episodes of graft rejection, mismatch in donor-recipient age, and smoking.

The purpose of this review article is the analyze current literature and find different anti-proliferative agents that can suppress the immune system and can thus contribute to the long-term survival of renal transplants. The findings of this review paper can be helpful in understanding the long-term survival of renal transplants and various ways to improve it.

## Introduction and background

**Figure 1:**
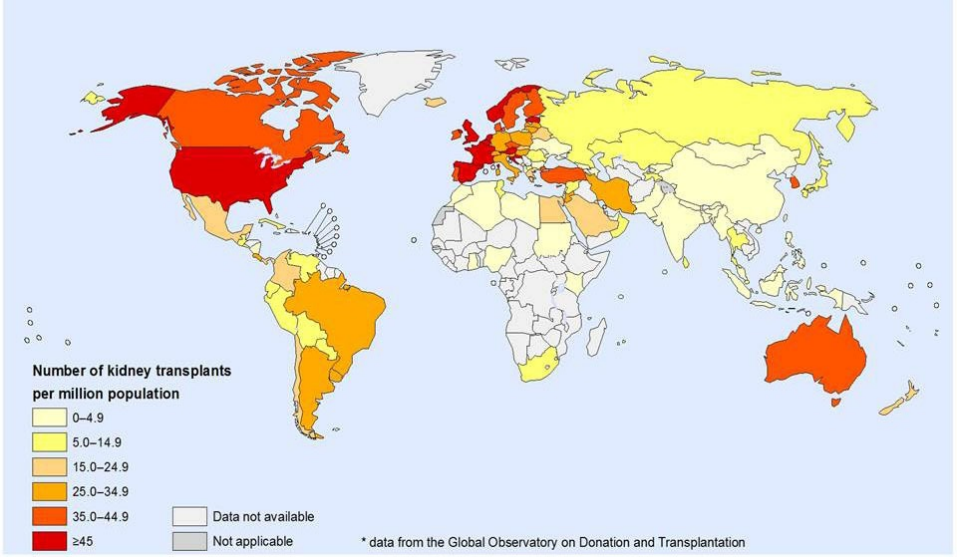
Kidney transplantation activities, 2012 Data from Global Observatory on Donation and Transplantation (GODT) data, produced by the WHO-ONT collaboration

**Figure 2:**
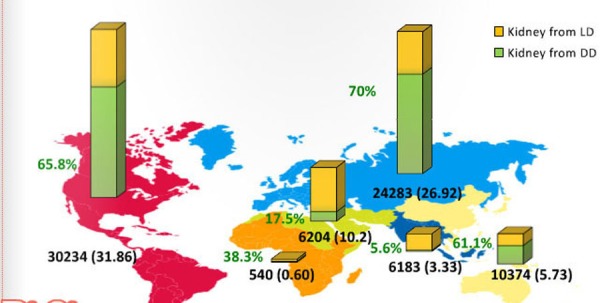
Kidney transplantation per region Data from Global Observatory on Donation and Transplantation (GODT) data, produced by the WHO-ONT collaboration

Kidney transplantation is the only effective treatment option for managing end-stage renal disease. According to statistics, 75,000 kidney transplants were done globally in the year 2010, and these statistics are expected to rise to 350,000 (almost three to four times the baseline value) in the coming years [1]. Figure 1 depicts the kidney transplantation activities of 2012; Figure 2 depicts the region-wise rate of kidney transplantation.

Transplant rejection is one of the biggest limitations in renal transplant procedures, where the kidney can undergo an acute, late acute, or chronic transplant rejection [2-3]. With the advancement in transplantation protocols, acute and long-term survival of renal transplants has improved [4], but long-term survival is still unsatisfactory. Acute transplant rejection has experienced a significant fall due to the use of immunosuppressant therapy, but most of the renal transplants develop chronic graft rejection within a decade [1, 5-6]. In addition to that, the long-term mortality rates among patients with renal transplants are observed to be significantly higher when compared to that seen in the general population [7]. Unfortunately, there is little we know when it comes to improving long-term survival of renal transplants. Therefore, time is of the utmost importance in understanding the underlying mechanism of long-term renal transplant rejection and explore different drug targets that can improve survival of both the graft and the patient.

## Review

A successful kidney transplantation is a much better option to improve the quality and longevity of chronic and/or end-stage renal disease patients. This is especially true when dialysis is the only other option. Dialysis procedure is very time-consuming, expensive, and requires frequent hospital visits. Kidney transplantation is thus a viable alternative. However, transplantation also has a fair share of disadvantages, the major constraint being renal transplant rejection.

### Histological and clinical features of chronic renal transplant rejection

Chronic renal transplant rejection is the result of a gradual decrease in the kidney function that starts to become evident three months after the transplantation surgery. Hypertension and proteinuria are the most important features of declining renal function [8-9]. Moreover, analysis of the serum creatinine concentration has shown that at least 80% of patients experience progressive loss of kidney function and start to exhibit signs of chronic allograft nephropathy (CAN) [10]. At least 50% of the patients with renal transplant develop features of CAN within 10 years of their transplant [11]. The major pathological features of CAN includes tubular atrophy, fibrous thickening of the arteries, fibrosis of the kidney interstitium, and glomerulosclerosis [12-13].

Transplant vasculopathy is the single most important feature of chronic renal transplant rejection [14]. Vasculopathy not only affects the large arteries but can also involve small peritubular capillaries [15]. The most important features of transplant vasculopathy include thickening of the fibrointima of the blood vessels, infiltration of the vessel walls with inflammatory cells, and breaks in the elastic layer of blood vessels. The subendothelial accumulation of smooth muscles in transplant vasculopathy was previously thought to be the result of migration of donor myofibroblasts from the media of the adjacent blood vessels. However, recent evidence has suggested that these smooth cells are from the recipient and are derived from the precursor cells present in the circulation [14-17]. The glomerular lesions seen in the biopsies obtained from the cases of CAN show wrinkling of the glomerular tuft of capillaries, focal glomerulosclerosis, hypertrophy of the glomeruli, and expansion of the mesangial matrix [18-20].

Transplant glomerulopathy can be distinguished from other forms of glomerulopathy like membranoproliferative glomerulonephritis (MPGN), based on the results of electron and immune-fluorescence testing. MPGN is characterized by electron-dense deposits, whereas the deposits seen in transplant glomerulopathy are electron lucent. Moreover, the immune deposits seen in MPGN patients are predominantly C3, whereas the main type of deposits in patients with transplant glomerulopathy is of the IgM type [21].

### Risk factors for chronic renal transplant rejection

The risk factors for chronic renal transplant rejection are described in Table 1.

**Table 1:** Comparison of the major risk factors governing survival of renal transplants

Factors for Increased Graft Rejection	Survival of the Graft	Factors for Better Graft Survival	Survival of the Graft
Acute transplant rejection episodes	6.6 years	No acute transplant rejection episodes	12.5 years
Non- HLA matched grafts	8.6 years	HLA matched graft	12.4 years
Recipient age <14 years	Less chances of <5 years survival	Recipient age >14 years but <70 years	More chances of >5 years survival
Donor-Recipient Mismatch (Young recipient-Old donor)	8.7 years	Donor-Recipient Match (Young recipient-Young donor)	11.64 years
Black Race	7.2 years	White Race	13.3 years
Antibodies to both class 1 and class 2 HLA antigens (2 years survival)	71%	Antibodies to either HLA class 1 or class 2 antigens (2 years survival)	77% and 79% respectively

### Acute transplant rejection episodes

The long-term survival of renal transplants is relatively shorter in those patients who experience episodes of acute transplant rejection as compared to patients who do not experience such episodes. The mean survival in patients with acute rejection patients vs. patients with no such episodes is 6.6 years and 12.5 years, respectively [22].

### Human leukocyte antigen (HLA) mismatching

Major histocompatibility complex (MHC) molecules on the transplanted kidney are the primary targets of the kidney recipient's immune system. Therefore, HLA matching can save a lot of transplants from rejection. The mean survival for the HLA-mismatched vs. HLA-matched transplants is 8.6 years and 12.4 years, respectively [23].

### Sensitization to HLA antigen

Pre-sensitization to Class 1 and Class 2 HLA is another important risk factor for chronic transplant rejection. Patients who are positive for the antibodies against Class 1 and Class 2 HLA usually have a poor long-term survival rate [24-26]. The two-year survival rate in individuals positive for anti-HLA Class 1 antibodies, anti-HLA Class 2 antibodies, and both Class 1 and Class 2 HLA antibodies is 77%, (vs. 84% anti-HLA Class 1 antibody-negative individuals), 79% (vs. 84% anti-HLA Class 2 antibody-negative individuals) and 71% (vs. no deleterious effects in individuals with a well-matched kidney), respectively [27].

### Recipient age

Younger recipients are more likely to suffer chronic renal transplant failure as compared to older individuals. Younger patients have a more responsive immune system and show poor compliance to immunosuppressive therapy [28-29]. A recent study has demonstrated that the five-year survival of grafts in younger children (< 14 years) is much lower when compared to that of individuals of other age groups [30].

### Match of donor-recipient age

Better matching of donor-recipient age is another important factor that can improve the outcomes of renal transplant. The mean survival of renal transplants in younger recipients receiving transplants from younger donors is 11.64 years as compared to the mean survival of 8.7 years in younger patients who receive grafts from older donors [31].

### Race

The chances of graft rejection are greater among blacks as compared to whites [32-33]. This difference is perhaps due to the difference in immune responsiveness among two groups [34]. The mean survival of grafts in blacks as compared to whites is 7.2 years and 13.3 years, respectively [4].

### Other risk factors

Other factors that may increase the risk of chronic renal transplant failure include loss of renal function [35], hypertension [36], proteinuria one-year post-transplantation [37], hyperlipidemia [38], and smoking [39].

### Pathophysiology of chronic renal transplant rejection

**Figure 3:**
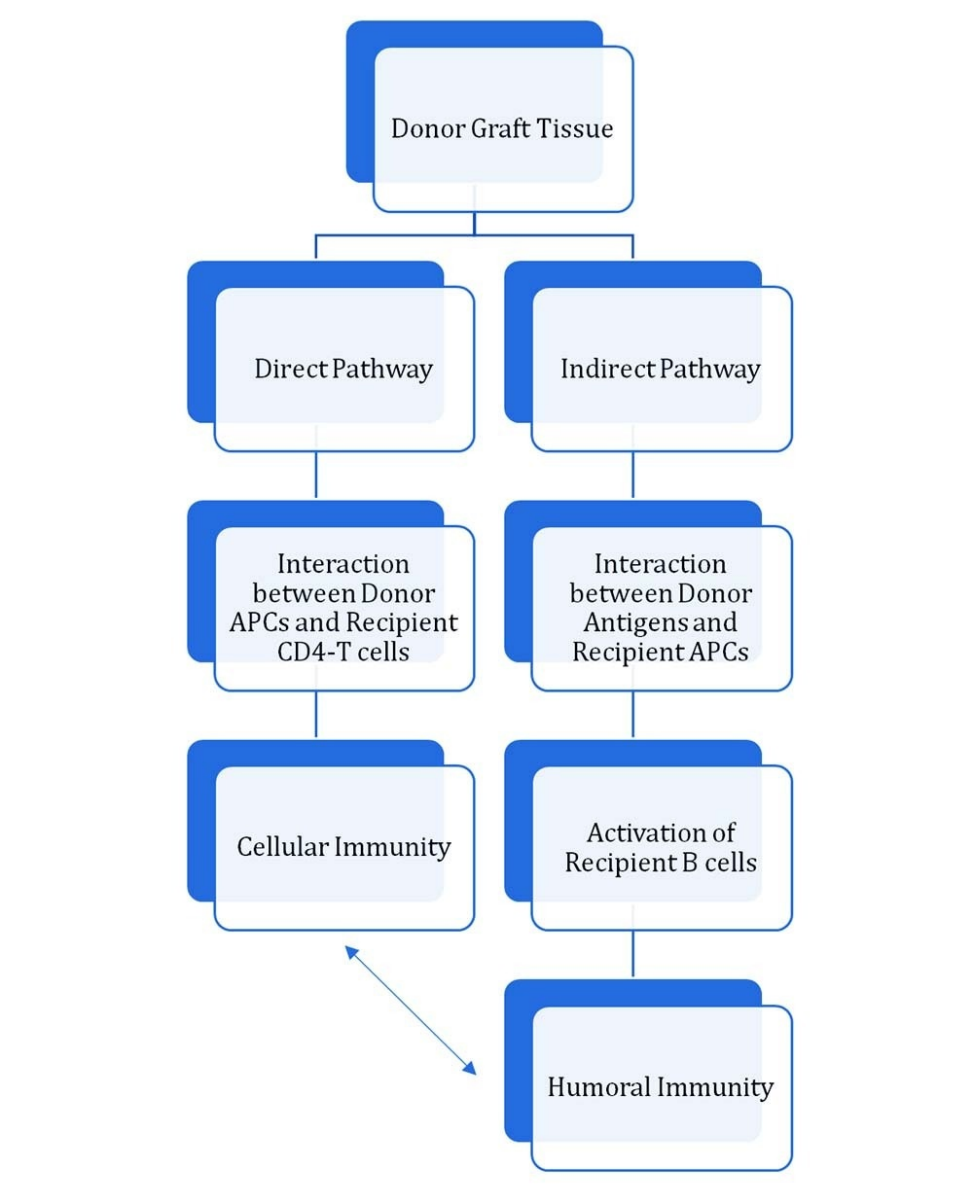
A scheme showing direct and indirect pathways for allograft rejection APCs: Antigen Presenting Cells

The fundamental component of chronic graft rejection is the detection of antigens on the donor’s tissues as “foreign entities” by the recipient’s immune system. However, the degree of immune reaction, and thereby degree and speed of graft rejection, depends on the histocompatibility between donor and recipient, as HLA matched grafts survive longer as compared to HLA-mismatched grafts [40-41]. The activation of the immune system involves two distinct pathways: the direct and the indirect pathway (Figure 3). The direct way involves the activation of CD4+ T cells by the donor’s antigen presenting cells (APC). The indirect pathway involves the processing of the donor’s graft antigens by recipient’s APC that then activates the immune cells [42]. The indirect activation can stimulate the activity of activated B cells, which then leads to the production of antibodies against the graft tissues. These antibodies seem to play a very important role in chronic graft rejection [43-44].

Cellular immunity has only a minor role in the chronic allograft rejection. Studies have shown that an indirect pathway is the main predictor for chronic allograft rejection [45-46]. The only role T cells play in chronic transplant rejection is via cytokines secreted by Type 2 helper lymphocytes (Th2). Several studies have shown that the cytokines secreted by Th2, like IL-4, IL-5, IL-6, IL-10, and IL-13, are responsible for reactions, such as tissue fibrosis and chronic rejection [47-48]. This fact has been further validated in studies where the injection of Th2 cells in immune-deficient recipients resulted in chronic graft rejection [49]. Blocking the effect of Th2-released cytokines can seemingly slow down the process of allograft rejection, which has been demonstrated through studies where the fibrosis in skin grafts was prevented via injection of anti-IL-4 antibodies [5]. The cytokines produced by Th2 cells causes allograft rejection through different ways. For instance, IL-4 stimulates the activity of fibroblast cells that increase the production of extracellular matrix and speed up the process of fibrosis. Similarly, a high titer of IL-10 inhibits the production of metalloproteinase by macrophages, the basic function of which is to digest excessive extracellular matrix [51-52]. Moreover, the cytokines produced by Th2 cells also promote the production of antibodies [53].

As previously mentioned, the basic role in allograft rejection - whether acute of chronic - is played by B cell and anti-HLA antibodies. The presence of plasma cells and CD20+ B cells in the allografts have been found to be associated with irreversible allograft injury [54-55]. The diagnosis of chronic (or acute) antibody-mediated allograft rejection is based on the presence of three different features: (i) presence of anti-donor antibodies, as indicated by serology, (ii) C4d, a complement split product, positive staining in the peritubular capillaries, and (iii) morphological features of chronic (or acute) renal tissue injury [56-57]. As already mentioned, cellular immunity accentuates humoral immunity, and humoral immunity accentuates cellular immunity. Humoral immunity damages the graft by the production of anti-graft antibodies via activation of T cells through an indirect pathway [58]. Moreover, stimulation of B cells, in the presence of T cells, drives the naïve B cells to differentiate into memory B and plasma cells, which provides long-lasting immunity against grafts [59-60].

### Drug targets for chronic renal transplant rejection

Different drugs that might be helpful in reducing chronic renal transplant rejection have been summarized in Table 2. Details of these drugs are as follows.

**Table 2:** Major drugs, their group, mechanism of actions and effects

Drug	Category	Mechanism	Effect
Mycophenolate mofetil	Immunosuppressive (Anti-proliferative)	Inhibitor of inosine monophosphate dehydrogenase (IMPDH)	Decreases proliferation of B and T cells.
Rapamycin (Sirolimus)	Immunosuppressive (Anti-proliferative)	Blocks Cells Cycle at the Junction of G1 and S phase by interacting with intracellular protein, FKBP12 and blocking cell specific kinase TOR (Target of rapamycin)	Decreases proliferation of B cells, T cells, smooth muscles and decreases antibody production
Everolimus	Immunosuppressive (Anti-proliferative)	Same as Rapamycin (Sirolimus)	Same as Rapamycin (Sirolimus)
Leflunomide	Immunosuppressive (Anti-proliferative)	Blocks the action of dihydroorotate dehydrogenase, which is a rate-limiting enzyme in the production of uridine monophosphate (UMP).	Decreases proliferation and differentiation of activated lymphocytes
Azithioprine	Immunosuppressive (Anti-proliferative)	Blocks de novo purine synthesis	Blocks T cell activation
Methylprednisolone	Immunosuppressive (Anti-proliferative and anti-inflammatory)	Causes redistribution of T cells and blocks inflammatory pathways	Decreases circulating T cells and inflammatory cytokines (for instance IL-6)
Tacrolimus (FK506)	Immunosuppressive (Anti-proliferative and antibiotic)	Causes decrease in gene expression	Decreases both cell-mediated and humoral immunity
Rituximab	Immunosuppressive (Anti-proliferative, anti-CD20 monoclonal antibody)	Antibody-dependent cellular cytotoxicity, direct signaling and antibody-mediated cytotoxicity	Decreases the population of CD20 B cells.

Mycophenolate Mofetil 

Mycophenolate mofetil (MMF) is a pro-drug for mycophenolic acid (MPA). It is an inhibitor of the inosine monophosphate dehydrogenase (IMPDH). It is an enzyme that controls the rate-limiting steps in the de novo production of guanosine nucleotides. T and B cells are more dependent on this pathway than any other cell [61-62]. Mycophenolate has been shown to have far more superior immunosuppressive properties as compared to azathioprine, and it can significantly reduce the chances of acute graft rejection [63]. However, its benefits in terms of long-term graft survival still need to be elucidated. Multiple studies have shown that the use of mycophenolate mofetil over an extended period can significantly reduce the chance of long-term graft rejection and can increase the mean survival of the transplant [64-65].

Rapamycin

Rapamycin (sirolimus), which was initially discovered as an antifungal agent, has now been shown to have significant anti-cancer and immunosuppressant activities. It blocks cell cycle at the junction of G1 and S phase by interacting with intracellular protein, FKBP12, and blocking cell specific kinase TOR (target of rapamycin) [66-67]. Several studies have shown that use of rapamycin in the maintenance regime after transplants can lead to immune suppression, decrease in smooth muscle proliferation, a decrease in the chances of acute and sub-acute transplant rejection, and improve the long-term survival and function of renal allografts [68-70].

Everolimus

Everolimus is a more polar version of rapamycin (sirolimus). Its mechanism of action and effects are essentially the same as rapamycin [71-73]. Data collected from pre-clinical studies have shown that everolimus not only improves the survival in response to acute graft rejection but also helps in the long-term survival of the grafts [72-74].

Leflunomide

Leflunomide is a drug that is widely used for the treatment of auto-immune disorders, like rheumatoid arthritis (RA), due to its potent immune-suppressive effects. The metabolites from leflunomide block the action of dihydroorotate dehydrogenase, which is a rate-limiting enzyme in the production of uridine monophosphate (UMP). Activated lymphocytes need UMP for proliferation and differentiation [75-77]. Leflunomide has been shown to decrease acute graft rejection [78-80]. Moreover, its use in animal models has been shown to decrease the development and progression of CAN in the transplanted tissues [81-82]. Results of a study showed that leflunomide was superior to azathioprine or mycophenolate mofetil in improving renal functions in transplant patients with deteriorating kidney functions. Moreover, its use decreased the progression and rather initiated the reversal of CAN features [83].

Azathioprine

Azathioprine is a purine analog that functions at the level of DNA [84-85]. It is quickly metabolized into 6-mercaptopurine (6-MP), which gets incorporated into the DNA and thereby decreases the *de novo* purine synthesis [86]. Azathioprine blocks CD28 signaling and T cell activation [87]. Studies have shown that shifting patients from the conventional cyclosporine A therapy to azathioprine therapy can improve the survival of graft and can decrease the chances of nephrotoxicity seen with cyclosporine [88].

Methylprednisolone

Methylprednisolone decreases the chances of chronic graft rejection by suppressing several immunological and inflammatory mechanisms. The exact mechanism by which the methylprednisolone accomplishes this feat is still uncertain, but two mechanisms are worth mentioning here. First, the administration of steroids causes the redistribution of T cells from the circulation into other body compartments (for instance, to bone marrow), which renders these cells almost ineffective [89-90]. Second, the administration of methylprednisolone also seems to decrease the production of inflammatory cytokines [91]. There are some reports that favor short, but not long-term, use of methylprednisolone as it reduces the chances of acute graft rejection and thereby, rather indirectly, improves the long-term survival of transplant patients [92].

Tacrolimus

Tacrolimus (FK506) is a macrolide antibiotic with immunosuppressive activity as well. Although its structure is different from cyclosporine, its mechanism of action is essentially the same as that of cyclosporine. It causes impairment in the expression of targeted genes in the targeted cells. Tacrolimus binds to an immunophilin, FK506 binding protein (FKBP), which then inhibits the activity of calcineurin phosphatase. Inhibition of calcineurin phosphatase suppresses the activity of several genes, such as genes involved in cell degranulation, interleukin-2 transcription, and so on. These effects of tacrolimus then inhibit the proliferation of T cells and their related cytokines. In addition, it also decreases the proliferation of B cells and antibody formation through an indirect effect, i.e. decrease in the activity of T cells, and suppresses the activation of B cells as well [93-94]. Tacrolimus has shown its efficacy over different conventional immunosuppressive agents in different clinical studies. It is less nephrotoxic as compared to cyclosporine and ensures the long-term conservation of kidney structure and function [95]. Moreover, it has an enhanced efficacy when used in combination with other immunosuppressive agents, such as MMF [96].

Rituximab

Rituximab is an anti-CD20 monoclonal antibody that significantly thins down the B cell population. Antibody-dependent cellular cytotoxicity, direct signaling, and antibody-mediated cytotoxicity are all the important pieces in its mechanism of action [97-99].There is some evidence that support the use of rituximab for improving the long-term survival of the kidneys [100-101].

## Conclusions

To conclude, the long-term survival of renal transplants is still poor. Different risk factors, like HLA-mismatching, acute episodes of rejection, mismatch of the donor-recipient age, the age of transplant, and race, contribute the most towards decreasing the long-term survival of kidneys. Moreover, both pillars of the immune system, i.e. cell-mediated immunity and humoral immunity, play a part in the rejection of kidneys in the long run. Therefore, improving the long-term survival of kidneys should include two important things. The first proactive step is to minimize the known risk factors before the actual renal transplantation. The second step is the usage of different anti-proliferative agents that can decrease the proliferation and action of immune cells to decrease the chances of graft rejection in the longer run. The choice of drugs for the same should be made only after vigilant consideration of multiple factors that are discussed in the review and should always be patient-specific.
